# The frozen-thawed sperm protein of Indonesian Toraya buffaloes is significantly associated with sperm kinematics, acrosome integrity, and mitochondrial membrane potential

**DOI:** 10.5455/javar.2024.k838

**Published:** 2024-12-27

**Authors:** Tulus Maulana, Syahruddin Said, Raden Iis Arifiantini, Jakaria Jakaria, Asep Gunawan

**Affiliations:** 1 Graduate School of Animal Production and Technology, Faculty of Animal Science, IPB University, Bogor, Indonesia; 2 Research Center for Applied Zoology, National Research and Innovation Agency, Bogor, Indonesia; 3 Division of Veterinary Reproduction and Obstetrics, School of Veterinary Medicine and Biomedical Sciences, IPB University, Bogor, Indonesia; 4 Department of Animal Production and Technology, Faculty of Animal Science, IPB University, Bogor, Indonesia

**Keywords:** CASA, frozen sperm, MMP, SDS-PAGE, Toraya buffalo

## Abstract

**Objective:**

The study aimed to characterize frozen-thawed sperm proteins and their correlation with sperm kinematics, acrosome integrity, and mitochondrial membrane potential (MMP) in Indonesian Toraya buffalo bulls.

**Materials and Methods:**

Semen was obtained from six Toraya buffaloes classified as Saleko and Bonga 4–10 years old. The frozen semen was analyzed for sperm motility, sperm kinematics, viability, abnormalities, membrane integrity, intact acrosome, MMP, DNA integrity, and sperm protamine (PRM) deficiency. Sperm protein concentration (Prot. Con) was determined by the bicinchoninic acid, and protein molecular weight (MW) was determined using 1D sodium dodecyl sulfate-polyacrylamide gel electrophoresis with 4%–20% gradient gel and 6.5–240 kDa protein marker.

**Results:**

The results of this study showed that the quality of frozen semen from Toraya buffalo bulls is in a good category and suitable for use in artificial insemination programs. The sperm quality differed significantly (*p < *0.05) between individuals, immunofluorescence examination of intact acrosome, PRM deficiency, intact MMP, and intact DNA showed no significant difference (*p < *0.05)*.* Pearson correlations in this study showed that sperm Prot. Con has a significant correlation (*p < *0.05) with acrosome integrity. The sperm protein band (Prot. Band) correlated significantly (*p < *0.05) with sperm kinematic parameters and intact MMP. The average sperm Prot. Con of Toraya buffalo was 77.29* ± *39.26 µg/ml and 4–13 Prot. Bands with 6–240 kDa of MW were detected, with Prot. Bands of 16, 50, 70, and 115 kDa having higher intensity.

**Conclusions:**

Frozen-thawed sperm protein is correlated with sperm kinematics, acrosome integrity, and MMP. The proteins were found to correlate with sperm quality and fertility in Toraya buffalo bulls.

## Introduction

Indonesia is a country known for its mega-biodiversity and abundance of genetic resources for livestock, including the Toraya buffalo (*Bubalus bubalis* Carabensis). These buffalo, found in Tana Toraja and North Toraja, South Sulawesi, have a distinctive spotted coat and are also phenotypically larger than swamp buffalo [1]. However, the population of Toraya buffalo is declining each year, mainly due to their use in traditional ceremonies. In addition, factors such as low reproductive performance and reliance on conventional breeding systems contribute to this decline [2]. To address these issues and improve the buffalo population and their performance in Indonesia, a planned artificial insemination (AI) program based on molecular data is necessary.

Superior bulls are selected for AI programs based on pedigree, production and reproductive ability, overall health, and freedom from disease, as determined by a breeding soundness evaluation method. However, assessing fertility in bulls is complex and requires various methods since no single objective test is sufficient. Molecular-based fertility tests, on the other hand, provide faster and more accurate results.

Research on the fertility of water buffalo bulls has identified several key factors that can predict in vivo fertility. Factors such as progressive sperm motility, rapid velocity, and average path velocity have shown positive correlations with fertility [3]. Singh et al. [4] discovered specific protein bands (Prot. Bands) in buffalo sperm that correlate with acrosome integrity and HOST positivity. This finding aligns with the results of Kikon et al. [5], who found that the addition of heparin-binding protein to frozen-thawed buffalo semen can affect the integrity of the acrosome and the mitochondrial membrane potential (MMP) of sperm.

Proteomic approaches have been utilized to identify potential biomarkers for bull fertility. Aslam et al. [6] observed overexpression of certain proteins, including PDZD8, CYP11B2, and ACRBP, in high-fertility sperm, while proteins such as MT1A, ATP5F1, and TUBB2B were more prevalent in low-fertility sperm. In addition, Fu et al. [7] reported high expression levels of proteins such as ODF2, AKAP4, and TUBB in highly fertile sperm. Singh et al. [4] identified 26 proteins significantly correlated with membrane integrity (M.I) and acrosome reaction, with proteins 55, 31, 26, 24, 20, and 18 kDa being potential fertility markers in buffalo sperm. Collectively, these studies indicate that a molecular protein approach can be employed to identify potential biomarkers for buffalo bull fertility.

Despite these advancements, there is a significant knowledge gap regarding the structural and functional properties of Toraya buffalo sperm. Understanding these molecular features is crucial, as they could potentially explain the observed low fertility in this breed. Currently, Toraya buffalo sperm proteins have not been thoroughly characterized, and the presence or absence of specific proteins could significantly impact sperm function and fertility. Because of the above facts and considering the lack of knowledge on Toraya buffalo sperm proteins and their correlation with sperm characteristics, this present study aims to separate the frozen buffalo bull sperm proteins using sodium dodecyl sulfate-polyacrylamide gel electrophoresis (SDS-PAGE) and determine the correlation between individual proteins and kinematic motility, acrosome status, and MMP.

## Materials and Methods

### Animals and ethical approval

**Figure 1.**
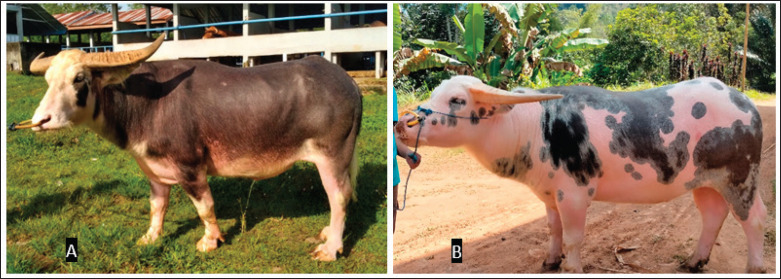
Type of coat spotted color pattern Toraya buffalo bulls in this study. *A-* Bonga tenge’; *B-* Saleko. (Source: personal images).

The semen used in the study was obtained from six Toraya buffalo bulls classified as Saleko and Bonga types shown in Figure 1. They were 4–10 years old and had an average body of 400–600 kg. The experimental designs and animal models used in this study were approved by the Animal Ethics Committee of the National Agency for Research and Innovation. Approval was granted under certificate number 050/KE.02/SK/03/2023.

### Semen collection and frozen semen preparation

The semen was collected through an artificial vagina and a female buffalo was used as a teaser. Semen was stored at 34°C and examined macroscopically and microscopically immediately after collection. Fresh semen with sperm motility of >60% was diluted with an Andromed extender. The commercial extender Andromed 1:4 were diluted with distilled water by following the instructions for use of the diluent on the package. The semen was diluted to 100 × 10^6^/ml and then filled into mini-straws with a volume of 0.25 ml (sperm concentration 25 × 10^6^ cell/straw).

The straw with the semen was placed in a freezing rack and equilibrated at 5°C for 4 h. The freezing rack was held 10 cm above the liquid nitrogen vapor for 15 min. The frozen semen was stored in liquid nitrogen containers at −196°C. After 24 h of storage, the frozen semen was thawed in a water bath at 37°C for 30 sec, both seals were opened, and the semen was placed in a microtube. During observation, the semen was stored in a dry bath at 37°C, and frozen semen was further analyzed for viability, morphology, sperm membrane, sperm intact-acrosome (SIA), MMP, DNA fragmentation index (DFI), and sperm protamine deficiency (SPD).

### Objective-based on motility and kinematics assessment

Sperm motility and kinematics were assessed using the computer-assisted semen analysis (CASA) system (SpermVisionTM 3.7.8 program (Minitube, Germany). The CASA was connected to Carl Zeiss Microimaging GmbH (Gottingen, Germany), which was equipped with a 38°C warm table. A total of 3 μl of semen sample was dropped onto a special chamber slide (Minitube, Germany). Subsequently, a total of 1600–2000 sperm cells were analyzed in four fields and were evaluated by factory setting for bull sperm with selected kinematics motility parameters shown in Table 1.

### Sperm viability, abnormality, and membrane integrity

Evaluation of sperm viability and abnormalities (ABNs) was performed using the eosin-nigrosine staining method. The assessment started by dropping 10 µl of semen, which was then mixed with 40 µl eosin-nigrosine (1:4) on the slide glass; both were homogenized, smeared, and dried at 37°C. The smears were viewed under a microscope with a magnification of 400×. Live (viable) sperm were not stained (transparent), while the dye solution stained non-viable sperm. Live and dead sperm were counted from 200 sperm cells. Sperm morphology was evaluated by viewing 200 sperm cells under a microscope with 400× magnification, distinguishing normal and abnormal sperm, and counting them.

The integrity of the sperm membrane was tested using the hypo-osmotic swelling (HOS) test by mixing 30 μl of semen into the microtube, containing 300 μl of HOS solution (0.735 gm of sodium citrate [Merck, Darmstadt, Germany], 1.351 gm of fructose [Merck, Darmstadt, Germany]; 100 ml of distilled water) then incubated in a dry bath (37°C) for 40 min, evaluation was carried out under a microscope with 400× magnification. The sperm that responded (coiled tail) or did not respond (straight tail) to the hypoosmotic solution were counted in 10 fields of view with a total of 200 cells.

### Sperm intact acrosome and protamine deficiency

The intact sperm-acrosome was assessed using the fluorescence staining method (Sigma, St. Luis, MO) combined with propidium iodide (P.I). Semen samples were made of smear, air-dried at room temperature, and fixed in 96% ethanol for 10 min. Subsequently, 30 μl (100 μg/ml) of peanut agglutinin (PNA) lectin solution was administered and incubated at 37°C for 30 min. Subsequently, 5 µl (1 µg/µl) of P.I (Sigma, St. Louis, MO) was applied to the smears and incubated for 5 min, then the smears were rinsed with phosphate-buffered saline (PBS). Acrosome status was assessed using a fluorescent microscope at a wavelength of 380–420 nm. A total of 200 sperm cells were observed in each sample. Sperm that exhibited a green fluorescent acrosome were categorized as intact, while sperm that exhibited a reddish color were considered damaged acrosomes [8].

**Table 1.** Selected parameters of sperm kinematics motility measured by CASA Spermvision.

Kinematic motility parameters	Description	Unit
Tmot	The complete total spermatozoa movement and assessed through Pmot and local motility	%
Pmot	The movement of sperm in a forward, straight line	%
VAP	The average velocity of the smoothed cell path The velocity along the average path of the spermatozoon	μm/sec
VCL	The average velocity of sperm along their actual curvilinear path	μm/sec
VSL	The average velocity measured in a straight line from the beginning to the end of the track	μm/sec
ALH	Mean width of the head oscillation as the sperms swim	μm
BCF	The frequency of the sperm‘s flagellar beat	Hz
LIN	The ratio of straight-line velocity over curvilinear velocity	%
STR	The ratio of straight line velocity over path velocity of the sperm‘s trajectory	%

**Figure 2.**
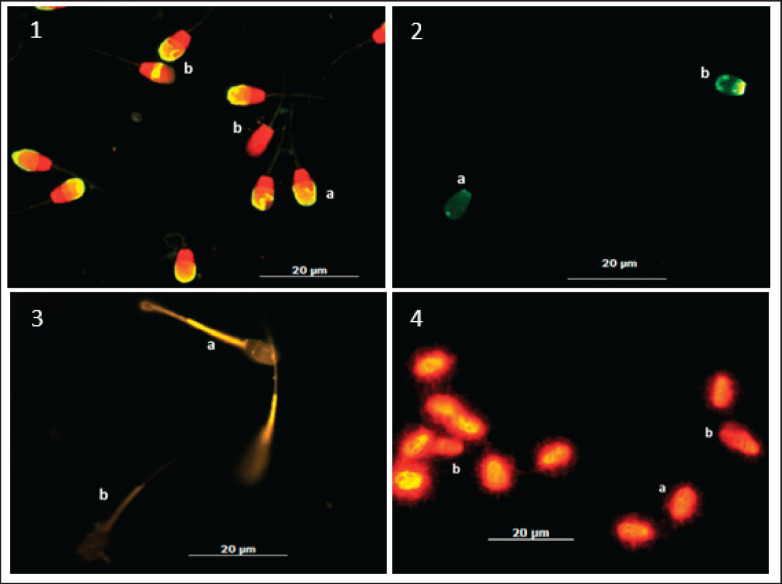
Magnification 63×, microscope fluorescence, Imager Z2, Carl Zeiss, Germany. *1-* Acrosomal status; *1a*- Intact acrosome (green fluorescence in the acrosome); *1b*- Non-intact acrosome (red fluorescence no acrosome); *2-* SPD; *2a*- Normal/complete PRM (dark/dull green fluorescence); *2b-* PRM deficiency (bright yellow fluorescence); *3-* Mitochondrial membrane potential (MMP); *3a-* Intact MMP (Yellow fluorescence in mitochondria); *3b*- No-intact MMP (dark/no fluorescence in mitochondria); *4-* Sperm DNA fragmentation; *4a*- Normal intact DNA (Small and intact halo); *4b-* Fragmented DNA (No halo or in-compact halo).

Frozen semen was thawed for 30 sec in a 37°C water bath. The semen sample was removed from the straw and placed in a microtube. During the experiment, the semen was kept in a 37°C water bath. The chromomycin A3 (CMA3) technique was used to test for PRM deficiency. The samples were washed twice in PBS by centrifugation and fixed in Carnoy’s solution for 8 min and centrifuged, then spread on slides rinsed with APES and airdried. All procedures were carried out at 4°C until the CMA3 treatment was performed. Each slide was stained in 100 µl of CMA3 solution (Sigma Chemical Co., St. Louis, MO) (0.25 mg/ml) in McIlvain buffer, pH 7.0, containing 10 mM MgCl for 30 min. The slides were then rinsed in McIlvain buffer and air-dried. The slides were viewed with a fluorescent microscope with a wavelength of 460–470 nm [8]. Sperm with PRM deficiency appear bright yellow, while sperm with good PRM content appear dark yellow/dull (Fig. 2).

### MMP examination using JC-1

Microscopic staining of sperms with JC-1 (Invitrogen) as per the manufacturer’s instruction. Sperm were incubated with 5 μM of JC-1 for 20 min at 37°C. Sperms were washed once with the appropriate medium and resuspended in a minimum volume to prepare the slide and observed under the fluorescence microscope at an excitation of 488 nm and emission at 590 nm for red/green fluorescence. At high MMP, JC-1 forms J-aggregates inside the mitochondria and emits orange/red fluorescence while in low MMP state, it remains in the monomeric form and emits green fluorescence/cloudy green (Fig. 2).

### Sperm DNA fragmentation assessment using Bos-halomax^®^ kit

Sperm DNA fragmentation analysis was performed using the Sperm-Bos-halomax^®^ kit (Halotech DNA, SL; Campusde Cantoblaco, Madrid, Spain) according to the manufacturer’s instructions. Following the procedure, the sample was stained with P.I, and the slide was incubated at 37°C for 5 min, followed by a rinse with PBS. After air-drying, 200 sperm cells were examined using a fluorescence microscope (Imager Z2, Carl Zeiss) with a 630× objective. The assessment involved observing for the presence of a large halo and dispersion, the absence of a halo or a cell showing no halo, indicative of sperm with DNA fragmentation, and a small, compact halo around the sperm head, representing sperm with intact DNA (Fig. 2).

### Sperm protein extraction and molecular weight determination by using SDS-PAGE

The frozen semen was washed using saline solution and centrifugated at 6,500 rpm for 30 min to separate sperm and extender. Following the manual’s instructions, the spermatozoa pellet was extracted using PRO-PREPTM Protein extraction solution (iNtRON Biotechnologi, Korea). The concentration of sperm protein was determined by the bicinchoninic acid protein assay (Thermo Scientific™, USA). SDS-PAGE analysis was performed to determine the protein profile based on molecular weight (MW), which is represented as bands on the gels. Protein separation was carried out using 1D SDS-PAGE using SurePAGE™, Bis-Tris, 10 × 8 cm, 12 wells, 4%–20% gradient gel (M00656; GenScript) (SurePAGE, Genscript Biotech Corp., Hongkong) with Broad Multi Color Pre-Stained Protein Standard (M00624; GenScript) with a MW range of ~6.5–240 kDa and Tris-MOPS-SDS Running Buffer (M00138; GenScript) with a voltage of 200 V and a current of 100 mA for 40 min. The gel was then stained using Coomassie Brilliant Blue (R-250; Bio-rad, USA).

### Statistical analysis

The data analysis of this study was performed with Statistical Minitab version 18.1 (Minitab for Windows, Minitab, Inc. USA). Due to the normal distribution and the different homogeneity of the data, the process was continued with the one-factor variance analysis (ANOVA) test. The Fisher test was then performed to analyze whether there were differences between the variables examined in the study. The correlation between frozen semen characteristics and sperm protein was analyzed with Pearson’s correlation using GraphPad Prism v.9.41 (GraphPad Software, USA). All study data were presented in the form of Mean *± *SD.

## Results

### Frozen-thawed sperm characteristics

The results of this study showed that the quality of frozen Toraya buffalo semen is in a good category and is suitable for use in AI programs. The sperm motility parameter in this study showed S1 bull had a significantly higher sperm motility (*p < *0.05), compared to the S5, and S6 bulls. The average sperm motility of all individual bulls was 53.58* ± *5.07%. The sperm viability parameter of S3 bull is significantly different (*p < *0.05), from bulls S3, S5, and S6, with an average sperm viability of all individual bulls being 53.56* ± *7.30%. For the sperm M.I parameter, the S3 bull differs significantly (*p < *0.05) from all buffalo bulls with the average sperm M.I of all individual bulls at 56.19* ± *6.70%. The percentage of sperm ABNs in S1 bull was lower and significantly different (*p < *0.05) compared to bulls S2, S3, S4, and S6. The mean sperm ABNs in this study is 7.86 *± *2.32% (Table 2).

Progressive motility (Pmot) in this study showed a significant difference (*p < *0.05) between individual bulls with an average of 43.13* ± *5.70%. The velocity parameters (curved velocity line [VCL]) of buffalo S2 bull also differed significantly (*p < *0.05) from those of S4 and S5 buffalo bulls with an average of 92.18* ± *7.33%. The kinematic parameters average velocity path (VAP), velocity straight line (VSL), linearity (LIN), LH, and beat cross frequency (BCF) on kinematics did not differ significantly (*p > *0.05) between bulls with an average of 64.66* ± *3.90%, 45.26* ± *1.69%, 44.46* ± *10.27%, 4.91* ± *0.55%, and 24.45* ± *1.13%, respectively (Table 3). Examination of sperm with intact acrosome, SPD, intact MMP, and intact DNA showed no significant difference (*p < *0.05) between individual Toraya buffaloes in this study, with a mean value of intact acrosome of 90.71* ± *2.96%, sperm normal PRM of 98.36* ± *0.57%, intact-MMP of 79.25* ± *4.56%, and sperm DNA integrity of 94.50* ± *1.82%, respectively (Table 4).

**Table 2.** Quality of frozen semen in various individuals of Toraya Buffalo Bulls.

Buffalo bulls	Sperm quality parameters (Mean *±* SD)			
	Motility (%)	Viability (%)	M.I (%)	ABN (%)
Bull 1 (S1)	61.32* ± *4.82a	56.31* ± *1.85ab	59.44* ± *4.32bc	4.86* ± *0.36c
Bull 2 (S2)	57.13* ± *4.31ab	42.99* ± *2.74c	52.49* ± *4.61cd	10.11* ± *1.95a
Bull 3 (S3)	51.52* ± *2.92bc	63.57* ± *3.57a	64.56* ± *1.25a	7.58* ± *0.72b
Bull 4 (S4)	54.3* ± *3.91abc	58.23* ± *1.20ab	61.9* ± *0.02bc	10.67* ± *0.55a
Bull 5 (S5)	49.7* ± *3.62bc	50.55* ± *9.74bc	48.27* ± *3.995d	6.31* ± *0.24bc
Bull 6 (S6)	47.56* ± *288c	49.21* ± *5.43bc	50.39* ± *9.39cd	9.76* ± *0.33a
Mean* ± *SD	53.58* ± *5.07	53.56* ± *7.30	56.19* ± *6.70	8.22* ± *2.34

Data show all Mean* ± *SD. Means in a column with different superscripts a, b, c, and d differ significantly at (*p < *0.05). M.I = membrane integrity.

**Table 3.** Kinematics (motion pattern) sperm motility of Toraya buffalo bulls.

Parameters	Buffalo bulls (Mean *±* SD)						Average
	Bull 1 (S1)	Bull 2 (S2)	Bull 3 (S3)	Bull 4 (S4)	Bull 5 (S5)	Bull 6 (S6)	
Pmot (%)	51.76* ± *4.99^a^	47.77* ± *3.44^ab^	39.75* ± *3.15^b^	42.86* ± *1.14^ab^	41.19* ± *6.38^b^	40.47* ± *3.85^b^	43.13* ± *5.70
VAP (µm/s)	65.14* ± *2.71^a^	70.67* ± *1.52^a^	64.59* ± *12.57^a^	60.29* ± *5.47^a^	60.59* ± *3.58^a^	66.74* ± *8.87^a^	64.66* ± *3.90
VCL (µm/s)	94.36* ± *5.30^ab^	104.59* ± *2.18^a^	88.72* ± *9.65^ab^	84.17* ± *9.70^b^	85.68* ± *9.16^b^	98.59* ± *8.81^ab^	92.18* ± *7.33
VSL (µm/s)	43.74* ± *1.51^a^	46.87* ± *0.10^a^	46.72* ± *8.46^a^	43.44* ± *0.72^a^	44.02* ± *3.40^a^	46.81* ± *7.69^a^	45.26* ± *1.69
STR (%)	66.50* ± *0.71^c^	66.00* ± *1.41^bc^	72.00* ± *1.41	72* ± *5.65^abc^	72.00* ± *1.41^ab^	69.5* ± *2.12^bc^	63.39* ± *14.44
LIN (%)	46.00* ± *1.41^ab^	44.50* ± *0.71^b^	51.5* ± *3.54^a^	51.5* ± *4.94^a^	51* ± *1.41^a^	49.5* ± *0.71^ab^	44.46* ± *10.27
ALH (µm)	5.31* ± *0.11^a^	5.77* ± *0.07^a^	4.84* ± *4.84^a^	4.36* ± *1.3^ab^	4.34* ± *0.67^ab^	4.89* ± *0.06^a^	4.91* ± *0.55
BCF (Hz)	23.39* ± *1.28^a^	22.93* ± *0.34^ab^	25.33* ± *3.06^a^	25.78* ± *2.11^a^	24.21* ± *3.56^a^	25.10* ± *4.06^a^	24.45* ± *1.13

Data show all Mean* ± *SD. Means in a row with different superscripts a, b, c, and d differ significantly at *p < *0.05. ALH = amplitude of lateral head deviation, BCF = beat cross frequency, LIN = linearity, STR = straightness, VAP = Average velocity path, VCL = curved velocity line, VSL = velocity straight line.

**Table 4.** Sperm intact acrosome, normal PRM, intact-MMP and intact DNA.

Buffalo bulls	Parameters (Mean *±* SD)			
	Intactacrosomal (%)	Normal PRM (%)	Intact-MMP (%)	Intact-DNA (%)
Bull 1 (S1)	88.30* ± *7.19	98.78* ± *0.36	81.26* ± *1.78	96.52* ± *2.06
Bull 2 (S2)	90.08* ± *1.04	98.86* ± *0.33	83* ± *8.48	96.20* ± *0.44
Bull 3 (S3)	87.71* ± *4.5	98.40* ± *1.27	76.68* ± *2.37	95.06* ± *3.62
Bull 4 (S4)	90.57* ± *0.67	97.85* ± *0.21	74.41* ± *1.78	93.22* ± *3.21
Bull 5 (S5)	95.98* ± *0.72	97.48* ± *1.43	75.15* ± *5.86	91.75* ± *3.88
Bull 6 (S6)	91.63* ± *3.35	98.75* ± *0.34	82* ± *1.41	94.23* ± *0.33
Mean* ± *sd	90.71* ± *2.96	98.36* ± *0.57	79.25* ± *4.56	94.50* ± *1.82

Data show all Mean* ± *SD. Means in a column with different superscripts a, b, c, and d differ significantly at (*p < *0.05). MMP = mitochondrial membrane potential.

### The correlation of sperm protein and sperm characteristics

**Figure 3.**
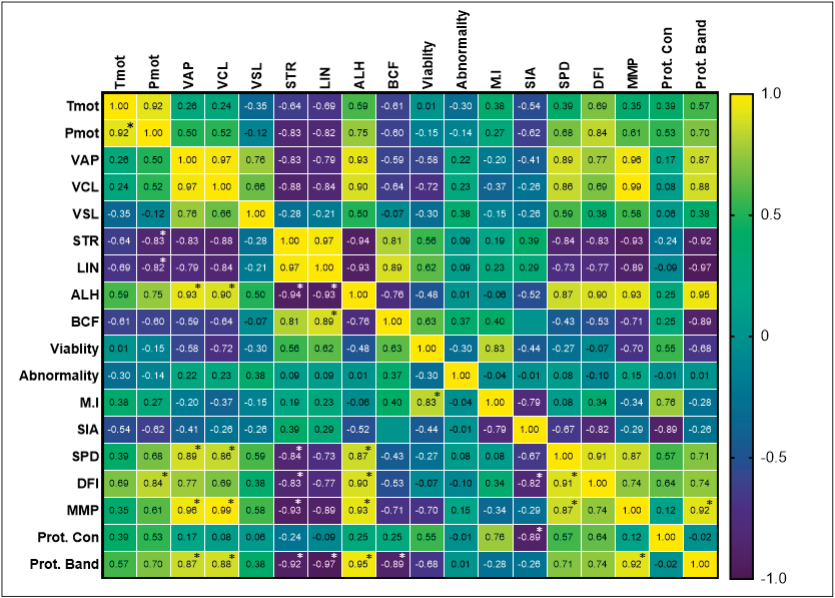
Heatmap visualization of Pearson’s correlations between Toraya buffalo sperm characteristics and sperm Prot. Con. The scale is based on colors from yellow (positive) to dark purple (negative). Value with an asterisk (*) has a significant correlation (*p < *0.05). ALH = amplitude of lateral head, BCF = beat cross frequency, DFI = DNA fragmentation index, LIN = linearity, M.I = membrane integrity, MMP = mitochondrial membrane potential, Pmot = progressive motility, Prot. Band = protein band, Prot. Con = protein concentration, SIA = sperm intact-acrosome, SPD = sperm protamine deficiency, STR = straightness, Tmot = total motility, VAP = average velocity path, VCL = curved velocity line, VSL = velocity straight line.

The results of the Pearson correlation in this study showed that sperm total motility (Tmot) has a significant positive correlation (*p < *0.05) with Pmot. In addition, kinematics parameters Pmot has a significant positive correlation (*p < *0.05) with sperm DFI. Subsequently, VAP correlates with amplitude of lateral head (ALH), SPD, and MMP. Similarly, VCL correlates with ALH, SPD, and MMP. Sperm viability shows a positive significant correlation (*p < *0.05) with M.I of 0.83, and sperm acrosome integrity correlates negatively with sperm DFI. On the other hand, there is a positive correlation between SPD with DFI and MMP (Fig. 3).

The results of the sperm protein concentration (Prot. Con) analysis are shown in Table 5. The S5 Bull has a lower Prot. Con of 32.09* ± *6.93 µg/ml was significantly different (*p < *0.05) compared to individual Bulls of S1, S2, S3, S4, and S6, while the average sperm protein of Toraya buffalo in this study was 77.29* ± *39.26 µg/ml. The results of the Pearson correlation in this study showed that sperm Prot. Con had a significant negative correlation (*p < *0.05) with sperm acrosome integrity parameters (−0.89). Subsequently, the sperm Prot. Bands showed a significant correlation (*p < *0.05) with the kinematic parameters VAP = 0.87, VCL = 0.88, ALH = 0.95, straightness (STR) = −0.92, LIN = −0.92, and BCF = −0.89 and sperm MMP = 0.92 (Fig. 3).

### The sperm MW of Toraya Buffalo

**Figure 4.**
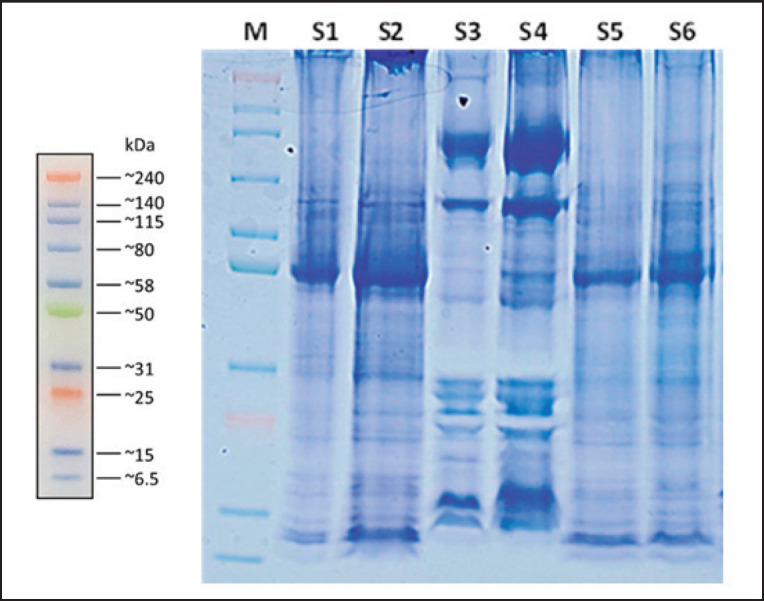
Sperm protein profile of individual Toraya buffalo bulls. M = marker; S1 = Bull 1; S2 = bull 2; S3 = bull 3; S4 = bull 4; S5 = bull 5; S6 = bull 6.

The results of MW determination by 1D-SDS page showed that the sperm Prot. Bands of Toraya buffalo in this study had 4–13 Prot. Bands with protein MWs of 6, 11, 15, 16, 23, 25, 26, 28, 30,45, 50, 53, 70, 80, 91, 115, and 240 kDa, respectively, showed in Table 6. The sperm protein samples from S3 and S4 bulls had the highest protein expression (13 Prot. Bands) compared to other samples, namely PRM1, transition protein 2 (TNP2), Coagulation factor XIII, Spermatogenesis associated 3 (SPATA3), Glutathione S- transferase (GST), Izumo sperm-oocyte fusion 1 (IZUMO1), Tubulin, HSP70, HSPB90b and Huntingtin-interacting protein 1 (HIP1), and as well as unknown protein at 240 kDa. In addition, S5bull had a lower number of Prot. Bands with four expressed Prot. Bands, Apolipoprotein C-III, TNP2, Coagulation factor XIII A chain, and Tubulin protein. The results also showed an abundance of proteins with high intensity in the Prot. Bands of 16, 50, 70, and 115 kDa, respectively, in each sample are shown in Figure 4.

**Table 5.** Sperm protein of Toraya Buffalo.

Buffalo bulls	Sperm Prot. Con (µg/ml)
Bull 1 (S1)	118.69* ± *24.56a
Bull 2 (S2)	70.76* ± *10.52ab
Bull 3 (S3)	108.98* ± *12.3a
Bull 4 (S4)	98.82* ± *38.62a
Bull 5 (S5)	32.09* ± *6.93bc
Bull 6 (S6)	95.03* ± *34.08a
Mean* ± *SD	77.29* ± *39.26

Data show all Mean* ± *SD. Means in a column with different superscripts a, b, and c differ significantly at (*p < *0.05)

## Discussions

### Frozen-thawed sperm characteristics

Sperm motility is a crucial factor in bull fertility because it directly affects the sperm’s ability to navigate through the female reproductive tract and reach the fertilization site. The mean sperm motility observed in this study is consistent with previous findings on indigenous Indonesian buffalo breeds, including Toraya buffalo [2], Aceh buffalo [9], Swamp buffalo [10], and Murrah buffalo [11]. These studies highlight the importance of evaluating sperm quality parameters such as motility, viability, M.I, and sperm ABNs.

**Table 6.** Electrophoretic profiles sperm protein of Toraya buffalo bulls.

MW (kDa)	Candidate protein	Bulls number						The overallpresence of protein *n* (%)
		S1	S2	S3	S4	S5	S6	
240	Unknown protein	-	-	+	-	-	-	1/7 (14.28)
115	HIP1	-	-	+	+	-	-	2/7 (28.57)
91	Heat shock 90 kDa protein (HSP90)	-	-	+	+	-	-	2/7 (28.57)
80	Centrosomal protein kizuna‐like	-	-	-	-	-	+	1/7 (14.28)
70	Heat shock 70 kDa protein (HSP70)	+	+	+	+	-	+	6/7 (85.71)
53	Cytochrome b-c1 complex subunit 1	-	-	-	-	-	+	1/7 (14.28)
50	β-tubulin protein	+	+	+	+	+	+	7/7 (100)
45	IZUMO1	-	+	+	+	-	-	3/7 (42.85)
30	SPACA1	+	+	+	+	-	+	5/7 (71.42)
28	ATP synthase complex subunit B1 mitochondrial	-	-	+	+	-	-	2/7 (28.57)
26	GST	-	+	+	+	-	-	3/7 (42.85)
25	SPATA3	-	+	+	+	-	+	4/7 (57.14)
23	Coagulation factor XIII A	+	+	+	+	+	+	6/7 (85.71)
16	Calmodulin	+	+	+	+	-	-	5/7 (71.42)
15	Nuclear TNP2	+	+	+	+	+	+	7/7 (100)
11	Apolipoprotein C-III	+	+	-	-	+	+	5/7 (71.42)
6-7	Sperm PRM P1	-	+	-	+	-	+	4/7 (57.14)
∑ Bands		8	11	13	13	4	10	

MW = molecular weight, , (+) = protein expressed, (-) = protein non-expressed, S1 = Bull 1, S2 = bull 2, S3 = bull 3, S4 = bull 4, S5 = bull 5, S6 = bull 6.

Statistical analysis showed significant differences (*p < *0.05) in the quality of frozen semen among individual Toraya buffaloes. The extreme temperature fluctuations during freezing, from 34°C to −196°C, can cause structural changes in sperm, especially in the plasma membrane, leading to reduced motility and increased DNA damage. Despite these challenges, the frozen semen from bulls S1 to S6 met the criteria for AI set by the Indonesian National Standard (SNI 4869-2:2021), with post-thaw motility ≥40% and sperm ABNs ≤20% [12].

Comparable post-thaw motility values have been reported in Kundhi, Murrah, and Jafarabadi buffaloes, with motility rates of 43.25* ± *3.40%, 41.67* ± *2.90%, and 57.60* ± *0.36%, respectively [13–15]. Sperm motility is regulated by intrinsic factors, including adequate levels of adenosine triphosphate (ATP), functional axonemal dynein ATPases, protein phosphatases, and an intact axoneme in a suitable ionic environment [16]. These factors are crucial for maintaining sperm motility and ensuring successful fertilization.

### Correlation of frozen-thawed sperm protein with sperm kinematic characteristics

This study found a statistically significant positive correlation (*p < *0.05) between sperm viability and M.I, highlighting the importance of a well-preserved membrane for maintaining cell function and preventing cellular damage. The kinematic data obtained from the Computer-Assisted Sperm Analysis (CASA) system provided a more precise and accurate assessment of sperm motility compared to conventional microscopic methods. Patel et al. [17] emphasized the advantages of CASA, including rapid and accurate evaluation of sperm concentration, motility, morphology, and kinematic characteristics. The mean kinematic motility in this study was lower than that reported for Jafarabadi, Mehsana, Thai Swamp, and Murrah buffaloes. In those studies, Pmot was >45%, VAP > 70 μm/s, VSL > 55 μm/s, VCL > 125 μm/s, STR > 60%, LIN > 40%, ALH > 6, and BCF > 23 Hz [15,17,18]. This variation is likely due to differences in buffalo breeds, sperm-freezing capabilities, and genetic quality. Maylem et al. [18] reported that cryopreservation significantly decreased sperm velocity and motility parameters, although a high percentage of moving cells were still observed post-thaw. Patel et al. [17] further noted significant positive correlations between Pmot and parameters such as VAP, VSL, ALH, and BCF.

In this study, kinematic motility showed a significant positive correlation (*p < *0.05) with sperm motility, intact sperm PRM, and MMP. An increase in MMP was associated with improved sperm motility and fertility potential. Paoli et al. [19] reported a positive correlation between Tmot and MMP (Δψ), suggesting that sperm motility depends on mitochondrial integrity.

Furthermore, a significant negative correlation (*p < *0.05) was observed between intact sperm acrosome and DNA fragmentation. The presence of an intact acrosome is essential for fertilization since it contains enzymes like hyaluronidase, which facilitate oocyte penetration [20]. The study found that sperm with intact acrosomes had a higher fertility potential, consistent with previous studies [21]. The correlation between SPD, DFI, and MMP suggests that changes in PRM levels may impact genomic and mitochondrial integrity, ultimately affecting male fertility.

PRM deficiency in sperm can be detected using the fluorescent dye CMA3, which competes with PRMs for binding to the DNA minor groove. This method is useful for evaluating sperm chromatin structure and identifying ABNs in chromatin packaging, which may indicate male infertility [22]. The study’s findings are consistent with previous research on sperm DNA integrity. For instance, Kumar et al. [15] reported that fertile bulls exhibited a DNA fragmentation percentage of 90.24* ± *0.94%, compared to 88.37* ± *0.91% in subfertile males. The observation of 10%–12% DNA fragmentation in frozen-thawed buffalo sperm suggests that apoptotic changes, including DNA fragmentation and phosphatidylserine translocation, may occur during spermatogenesis.

### MW of sperm proteins in Toraya buffalo

This study is the first investigation into the relationship between sperm protein content and sperm Prot. Bands with frozen semen quality in Toraya buffalo bulls. The sperm Prot. Con in Toraya buffalo was found to be lower than that reported by Dixit et al. [23] in Bharadai buffalo (4.42* ± *0.63 mg/dl). The 1D-SDS-PAGE analysis revealed 4–13 Prot. Bands in Toraya buffalo sperm, consistent with Dixit et al. [23], who identified 14 Prot. Bands (16–205 kDa) in Bharadai buffalo. In contrast, Singh et al. [4] found 18 Prot. Bands (18–135 kDa) in frozen Murrah buffalo sperm.

The 240 kDa Prot. Band, expressed in bull S3 sperm, was identified as a component of a larger molecule and correlated with high sperm viability, M.I, and concentration. Capkova et al. [24] reported that the 240 kDa protein is a key component of the cyclic nucleotide-gated channel in rod photoreceptor cells, which plays various roles in human sperm function, including adhesion, motility, and fertility. However, further investigation is needed to determine the specific role of the 240 kDa protein in sperm function and fertility.

Several highly expressed proteins were identified in this study, including HIP1, expressed in bulls S3 and S4. Rao et al. [25] reported that HIP1 plays a critical role in spermatogenesis, particularly in the differentiation, proliferation, and survival of spermatogenic progenitors. The HSP90 protein was expressed in bulls S3 and S4 and has been found in the midpiece of sperm in various species, including humans. The higher expression of HSP90 could enhance motility and freezing resistance [26].

HSP70 protein was present at high intensity in all bull sperm, except in bull S5. This observation correlates with the lower sperm M.I and Prot. Con observed in S5. A study on bull fertility concluded that sperm motility positively correlates with HSP70 expression levels and that sperm acquire HSP70 through epididymal secretion. HSP70 is involved in various sperm functions, including motility, energy metabolism, protein processing, and male fertility [27].

The β-tubulin (50 kDa) protein was also expressed with high intensity in all buffalo bulls’ sperm. The β-tubulin protein is a crucial component of the sperm tail’s microtubules, essential for motility and fertility. Studies have linked human sperm centrosomal proteins, including tubulin, to fertility, highlighting the importance of tubulin in sperm function and male fertility [28]. The calmodulin (16 kDa) protein was expressed with high intensity in bulls S1–S4. Lasko et al. [29] reported that calmodulin, a calcium-binding protein, regulates sperm functions such as motility and fertilization. Proteins such as IZUMO, sperm acrosome membrane-associated protein 1 (SPACA1), GSTs, SPATA3, TNP2, and PRM1 are important for understanding sperm quality, male fertility, and fertilization. Research indicates that IZUMO1 is crucial for sperm-egg fusion and mammalian fertilization, while SPACA1 is involved in acrosomal morphogenesis and sperm-egg binding. GSTs protect sperm from oxidative stress, SPATA3 is involved in spermatogenesis and motility, TNP2 plays a critical role in chromatin remodeling during spermatogenesis, and PRM1 protects sperm DNA from damage and is vital for normal sperm function in various species. Although ApoC-III is not directly connected to sperm, other apolipoproteins, and proteins related to lipid metabolism may impact male reproductive health [30].

While this study provides valuable insights into the characteristics of frozen-thawed sperm from Toraya buffalo, there are a few limitations. First, the sample size was limited to a specific population of Toraya buffalo, so it may not fully represent the genetic diversity of the breed or apply to other buffalo breeds. In addition, although advanced techniques like CASA and 1D-SDS PAGE were used in the study, the interpretation of kinematic and protein data could be strengthened by incorporating more sophisticated proteomics approaches, such as liquid chromatography-mass spectrometry, to allow for more precise identification and quantification of sperm proteins involved in fertility. Finally, since the study was observational, it did not establish a causal relationship between sperm protein expression and fertility outcomes. Therefore, further experimental studies are needed to explore these associations more definitively.

## Conclusion

The results of this study suggest that the frozen-thawed sperm protein has a significant correlation with sperm kinematics, acrosome integrity, and MMP. The evaluation parameters of frozen-thawed sperm characteristics by MW analysis (SDS-PAGE) can be used to predict semen quality and fertility; on the other hand, they can be used for the selection of Toraya buffalo bulls in Indonesia.

## List of abbreviations

HOST, Hypoosmotic Swelling Test; APES, Aminopropyltriethoxysilane; JC-1, 5,5’,6,6’-Tetrachloro-1,1’,3,3’-tetraethylbenzimidazolylcarbocyanine iodide; ANOVA, Analysis of Variance; LH correction ALH, Amplitude of lateral head; CASA, Computer-Assisted Sperm Analysis;

ABN, abnormality; AI, artificial insemination; ALH, amplitude of lateral head; ATP, adenosine triphosphate; BCF, beat cross frequency; CMA3, chromomycin A3; DFI, DNA fragmentation index; DFI, DNA fragmentation index; GSTs, Glutathione S-transferases; HIP1, huntingtin-interacting protein 1; HOS, hypo-osmotic swelling test; IZUMO1, izumo sperm-oocyte fusion 1; LIN, linearity; M.I, membrane integrity; MMP, mitochondrial membrane potential; MMP, mitochondrial membrane potential; MW, molecular weight; P.I, propidium iodide; PBS, phosphate-buffered saline; Pmot, progressive motility; PNA, peanut agglutinin; PRM, protamine; Prot. Band, protein band; Prot. Con, protein concentration; SDS-PAGE, sodium dodecyl sulfate-polyacrylamide gel electrophoresis; SIA, sperm intact-acrosome; SPACA1, sperm acrosome membrane-associated protein 1; SPATA3, spermatogenesis associated 3; SPD, sperm protamine deficiency; STR, straightness; Tmot, total motility; TNP2, transition protein 2; VAP, average velocity path; VCL, curved velocity line; VSL, velocity straight line.
